# Effects of Climate on Phthisis

**Published:** 1843-07-22

**Authors:** 


### EFFECTS OF CLIMATE ON PHTHISIS.

At the Academy of Medicine, Paris, M. Rayer read a report on a memoir by M. Boudet on the effects of climate in cases of pulmonary consumption.

The author had asked the following question :—Is phthisis a rare disease in Algiers, and is it much more rare in marshy countries than in other localities?

A few remarks will serve to point out the importance of this question. In an interesting memoir, recently addressed to the Academy, M. C. Broussais showed that the proportion of deaths from phthisis to the general mortality in the African army was as 1 to 102; whereas, in the French army, the deaths from this disease form one-fifth of the general mortality.

But, it may be asked, is this very great difference apparent or real?—May we not explain the slight mortality from phthisis by the great mortality of certain other affections?—Are the inhabitants as free from this affection as the military? Does the law hold good for all parts of Algeria.

Numerous and important reports have been forwarded by the military surgeons attached to the African army, but they do not enable us to solve the above question in a complete manner. M. Boudin, one of the most distinguished military surgeons, thinks that the rare occurrence of phthisis in Africa is not a general fact, but is confined to the marshy border of the sea coast, where intermittent fevers and other malarious disorders prevail. Hence, M. Boudin, and those who adopt his opinion, say that two conditions should be attended to in the choice of a climate for the consumptive—viz., a mild temperature, and a marshy (but not insalubrious) soil.

The facts adduced by M. Boudin in support of this opinion are worthy of notice.

The climate of Hyeres has been long regarded as favourable to the consumptive, and it is known that the prevailing diseases of the place are those common to marshy situations.

Pisa, Parma, Rome, a residence in which is so generally recommended to the phthisical, are exposed to marsh miasmata, and subject to intermittent fevers. Dr. Herineu, who passed eight years in the Ionian Islands, informs us that the rarity of phthisis is in direct ratio to the frequency of intermittent fevers; and M. Roux, a distinguished military surgeon, affirms the same relative to our own soldiers on the shores of the Morea.

Broussais says that, in the neighbourbood of Cadiz, inflammation of the chest and tubercles are extremely rare, the prevailing diseases consisting of abdominal affections and intermittent fevers. Dr. Green, of New York, affirms that at Whitehall, near Washington, where marsh fevers abound, consumption does not exist amongst the inhabitants, and that the phthisical patients who resort there are greatly and permanently benefitted.

From these and various other considerations the reporters think it right to advise that a statistical investigation should be made relative to the frequence of phthisis in dry and marshy situations, both in France, the North of Europe, and Africa. M. Nepple has shown that phthisis is rare in the marshy country about Bresse. M. Schoenlein assures us that, in the delta of the Rhine, at Amsterdam, Rotterdam, and in all the lower parts of Holland, where ague is endemic, tubercular affections are rare, while they are frequent in the dry sandy localities about Bruxelles, &c.—*Prov. Med. Journ.*

